# Changes in Tyrosine Hydroxylase Activity and Dopamine Synthesis in the Nigrostriatal System of Mice in an Acute Model of Parkinson’s Disease as a Manifestation of Neurodegeneration and Neuroplasticity

**DOI:** 10.3390/brainsci12060779

**Published:** 2022-06-14

**Authors:** Anna Kolacheva, Leyla Alekperova, Ekaterina Pavlova, Alyona Bannikova, Michael V. Ugrumov

**Affiliations:** Koltzov Institute of Developmental Biology, Russian Academy of Sciences, 26 Vavilova Street, 119334 Moscow, Russia; al.alekperova@gmail.com (L.A.); guchia@gmail.com (E.P.); mukurokun354@gmail.com (A.B.); michael.ugrumov@mail.ru (M.V.U.)

**Keywords:** Parkinson’s disease, MPTP model, mice, tyrosine hydroxylase, phosphorylation, neurodegeneration, neuroplasticity

## Abstract

The progressive degradation of the nigrostriatal system leads to the development of Parkinson’s disease (PD). The synthesis of dopamine, the neurotransmitter of the nigrostriatal system, depends on the rate-limiting enzyme, tyrosine hydroxylase (TH). In this study, we evaluated the synthesis of dopamine during periods of neurodegradation and neuroplasticity in the nigrostriatal system on a model of the early clinical stage of PD. It was shown that the concentration of dopamine correlated with activity of TH, while TH activity did not depend on total protein content either in the SN or in the striatum. Both during the period of neurodegeneration and neuroplasticity, TH activity in SN was determined by the content of P19-TH, and in the striatum it was determined by P31-TH and P40-TH (to a lesser extent). The data obtained indicate a difference in the regulation of dopamine synthesis between DA-neuron bodies and their axons, which must be considered for the further development of symptomatic pharmacotherapy aimed at increasing TH activity.

## 1. Introduction

The nigrostriatal system is a part of the basal ganglia; it is involved in the regulation of motor function and motor memory. The main neurotransmitter in this system is dopamine (DA), which is synthesized from tyrosine by two enzymes: tyrosine hydroxylase (TH) and aromatic L-amino acid decarboxylase (AADC). It is believed that the rate-limiting stage of DA synthesis is the formation of L-DOPA from tyrosine by TH; BH4 and Fe act as co-factors [1,2,3]. DA synthesized in the cytosol is taken up into vesicles by the vesicular monoamine transporter type 2 (VMAT2). DA is released from the DA axon terminals via vesicle exocytosis in response to an action potential. After action on the receptors of target neurons, DA is degraded by catechol-O-methyl transferase (COMT) and monoamine oxidase (MAO) contained in the intercellular space, glial cells, and axon terminals [4,5,6] or recaptured into the synaptic terminal with a dopamine transporter (DAT) and then into vesicles with VMAT2 for reuse. In the normal striatum, DA reuptake prevails over its degradation [7].

Parkinson’s disease (PD) develops in the process of degradation of the nigrostriatal system. PD is the second most common neurodegenerative disease that affects 1–3% of the world’s population over 60 years old [8]. The appearance of motor symptoms typical for PD is preceded by a long-term latent degradation of the nigrostriatal system, up to the degeneration of 50–60% of DA neuron bodies and a decrease in DA to 20–30% in the striatum (threshold values) [9]. In this case, the depletion of neuroplasticity in the brain also occurs [10,11,12,13]. One of these compensatory mechanisms may be an increase in the functional activity of surviving DA neurons. Nagatsu and coworkers showed that in PD, despite a decrease in the total content of TH and its activity, homospecific activity (TH activity per enzyme amount) increases [14,15].

TH regulation can be divided into fast, which includes post-translational changes of the protein (phosphorylation, inhibition by catecholamines, etc.), and slow, which is transcription activation [16,17]. The key post-translational process that increases the activity of TH is its phosphorylation at serine at positions 8, 19, 31, and 40. It has been shown that phosphorylation at positions P31-TH and P40-TH increases TH activity, and phosphorylation at position P19-TH increases the availability site 40 for phosphorylation (P40-TH). The role of phosphorylation at position P8 (P8-TH) is not completely clear [16,17,18,19,20,21,22]. It has also been shown that the phosphorylated protein at positions P19-TH is a marker of its degradation [18,23].

The regulation of TH activity by phosphorylation has been studied in various PD models in vivo, e.g., using neurotoxic models with the administration of 1-methyl-4-phenyl-1,2,3,6-tetrahydropyridine (MPTP) [24,25,26] and 6-hydroxydopamine (6-OHDA) [27]. However, in previous studies, the evaluation of TH activity and/or the content of its phosphorylated forms was performed without taking into account the progressive degradation of the nigrostriatal system. It seems important to study the regulation of TH during the period of neurodegeneration considering the peculiarities of the pathogenesis of the disease, where death of DA neurons is a permanent process. This will also allow to find new approaches to the development of symptomatic pharmacotherapy aimed at replenishing the level of DA in the striatum in PD.

Earlier in our laboratory, we developed a mice model of PD. The model uses four-fold administration of MPTP, which leads to the development of an early clinical stage in key parameters: the presence of impaired motor function, a decrease in the concentration of DA in the striatum and number of DA neuron bodies in the substantia nigra (SN) to a strictly defined threshold value [28]. This model was used to estimate the period of degradation of the nigrostriatal system not only in neuron bodies in the SN, but also in their axon terminals in the striatum [29,30]. Based on these studies, we chose a period of neurodegradation on DA neurons and a period when neuroplasticity begins to develop in the surviving DA neurons.

The goal of this study was to evaluate the synthesis of DA in DA neurons during the period indicated above, with an emphasis on the molecular mechanisms responsible for the change in TH activity. We evaluated the activity of TH, the content of TH and its phosphorylated forms (P19-, P31-, and P40-TH) in the striatum and SN, as well as the concentration of DA in the striatum during the degradation of the nigrostriatal system and a few hours after its completion.

## 2. Materials and Methods

### 2.1. Animals and Experimental Procedures

Male mice C57BL/6 aging 8–12 weeks and weighing 22–26 g (*n* = 130) were used in this study. The animals were housed at 21–23 °C in a 12 h light–dark cycle with free access to food and water. The experimental procedures were carried out in accordance with the National Institutes of Health Guide for the Care and Use of Laboratory Animals (8th edition, 2011) and were approved by the Animal Care and Use Committee of the Koltzov Institute of Developmental Biology of the Russian Academy of Sciences (protocol No. 44 from 24 December 2020 and No. 55 from 9 December 2021).

### 2.2. Modeling of the Early Clinical Stage of Parkinson’s Disease

An early clinical PD model was reproduced in mice with four subcutaneous injections of MPTP (Sigma Aldrich, USA) at a dose of 12 mg/kg of body weight (free base) in saline with 2 h intervals. The control animals received saline only [28,29].

### 2.3. Design of Experiments 

**Figure 1:**
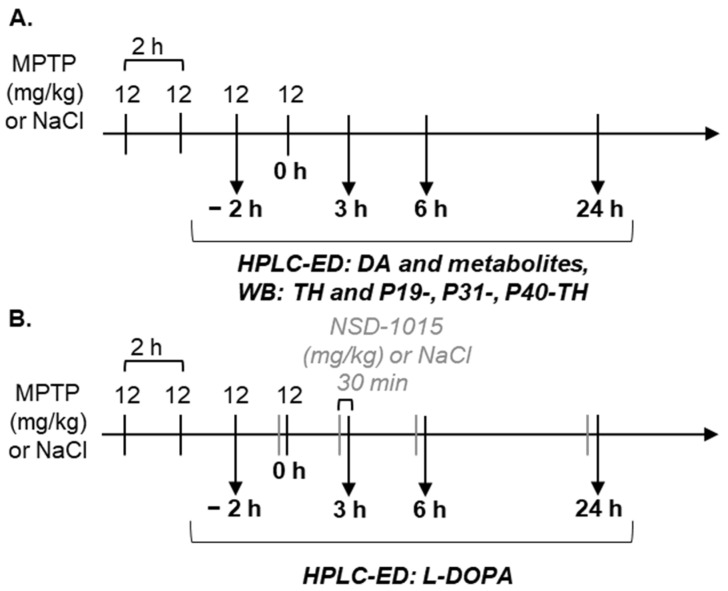
The scheme of the experiments for assessing: (**A**) the concentration of DA in the striatum as well as the content of TH and its phosphorylated forms at serine at positions P19-, P31-, and P40-TH and (**B**) the activity of TH during inhibition of DAA in the striatum and substantia nigra during the period of neurodegeneration (from −2 h to 6 h after MPTP injections) and neuroplasticity (24 h) in the nigrostriatal system in an early clinical PD model. DA—dopamine, HPLC-ED—high-performance liquid chromatography with electrochemical detection; L-DOPA—L-3,4-dihydroxyphenylalanine; MPTP—1-methyl-4-phenyl-1,2,3,6-tetrahydropyridine; NSD-1015—3-hydroxybenzylhydrazine; TH—tyrosine hydroxylase; SN—substantia nigra, WB—Western blot.

The scheme of experiments is shown in Figure 1.

In the first series of experiments, the content of TH and its phosphorylated forms in the striatum and SN was determined 3, 6, and 24 h after the fourth injections of MPTP (*n* = 8–10 in each group). Taking into account that the degradation of the terminal DA axon begins earlier than that of the cell bodies of DA neurons, the striatum was also isolated 2 h after the second MPTP injections (shown as −2 h in the diagram). In addition, for all indicated time intervals, the concentration of DA in the striatum was assessed.

The animals were anesthetized with isoflurane and decapitated. The striatum and SN were excised from the brain according to previously described methods [28,31]. All samples were weighed, frozen in liquid nitrogen, and stored at −70 °C until the analysis. The striatum from one half of the brain was used to determine the concentration of catecholamines by high-performance liquid chromatography with electrochemical detection (HPLC-ED). The striatum from the remaining half of the brain and the SN from both halves of the brain were used to determine the content of TH and its phosphorylated forms by Western blotting (WB). 

In the second series of experiments, TH activity was determined. Half an hour before the decapitation (2 h after 2 injections of MPTP and 3, 6, and 24 h after 4 injections of MPTP) mice of both experimental and control groups (*n* = 7–10 in each group) were i.p. administered with an AADC inhibitor 3-hydroxybenzylhydrazine (NSD-1015, Sigma Aldrich, USA) at a dose of 100 mg/kg of body weight [32]. After anesthesia and decapitation, the striatum and SN were excised from the brain, weighed, frozen in liquid nitrogen, and stored at −70 °C until analysis of the level of L-3,4-dihydroxyphenylalanine (L-DOPA) by HPLC-ED.

### 2.4. Sample Analysis

#### 2.4.1. High-Performance Liquid Chromatography with Electrochemical Detection 

In brain tissue samples, the concentration of DA and its metabolites (3,4-dihydroxyphenylacetic acid (DOPAC), 3-methoxytyramine (3MT), homovanillic acid (HVA)) or L-DOPA after DAA inhibition with NSD-1015 was determined by HPLC-ED. The samples were homogenized with a Labsonic M ultrasonic homogenizer (Sartorius, France) in 0.1 *n* HClO_4_ (Sigma Aldrich, USA) with 250 pmol/mL internal standard 3,4-dihydroxybenzylamine hydrobromide (Sigma Aldrich, USA). After that, the solution was centrifuged for 20 min at 2000× *g*.

HPLC separation was carried out on a reversed-phase column ReproSil-Pur, ODS-3, 4 × 100 mm with a pore diameter of 3 µm (Dr. Majsch, Germany) at +30 °C and a mobile phase speed of 1 mL/min supported by a liquid chromatograph LC-20ADsp (Shimadzu, Japan). The mobile phase consisted of 0.1 M citrate-phosphate buffer, 0.3 mM sodium octanesulfonate, 0.1 mM EDTA, and 8% acetonitrile (all reagents from Sigma Aldrich, USA), pH 2.5. An electrochemical detector Decade II (Antec Leyden, The Netherlands) was equipped with a working glassy carbon electrode (+0.85 V) and an Ag/AgCl reference electrode. Peaks of interest and internal standard were identified by their release time in the standard solution. The monoamine concentrations were calculated by the internal standard method using a calibration curve with LabSolutions software (Shimadzu, Japan). The concentration of DA was calculated according to the following equation:

The concentration of DA = (the area of DA peak in the sample solution/the area of DA peak in the standard solution) × (the area of DHBA peak in the standard solution/the area of DHBA peak in the sample solution (250 pmol/mL for striatum or 50 pmol/mL for SN) × concentration standart solution (250 pmol/mL for striatum or 50 pmol/mL for SN) × V,

where V is the volume of sample solution: SN—0.120 mL, striatum—0.400 mL. Striatal samples were normalized to tissue weight. The concentration of DA metabolites was calculated in a similar way. Retention time of DA and its metabolites was: DHBA—3.0 min, DOPAC—3.8 min, DA—4.3 min, HVA—8.7 min, 3MT—9.7 min.

The DA turnover was defined as the ratio of DA metabolites (DOPAC, 3MT, or HVA) to DA.

#### 2.4.2. Western Blot 

Striatal and SN samples from each experimental and control group were used for the Western blot assay. Tissue samples were homogenized in a RIPA buffer with a ×2 protease inhibitor cocktail (Thermo Fischer, USA) and a ×1 phosphatase inhibitor cocktail (Cell Signaling, USA). To the striatum and SN were added 300 and 140 µL of the buffer, and the samples were centrifuged at 20,000× *g* for 20 min. Then, the protein concentration was determined using a Bicinchoninic Acid Solution (BCA) as a protein assay test [33]. The samples were denatured at 95 °C for 5 min in Laemmli sample buffer consisting of 2% SDS, 10% glycerol, 5%-mercaptoethanol, 62.5 mM Tris (pH 6.8), and 0.004% bromophenol blue. Protein extracts were separated by electrophoresis in a 12% polyacrylamide gel with SDS in a buffer and transfered overnight to a nitrocellulose membrane (Hybond-enhanced chemiluminescence, Amersham Biosciences, USA). Equal loading was reconfirmed by Ponceau-S staining of each Western blot lane on the membrane [34,35]. The Ponceau-S was then removed by washing with a TBS buffer. The blots were blocked in TBS with 0.05% Tween 20 and 5% bovine serum albumin (BSA) for 1 h at RT and incubated with mouse monoclonal antibodies to TH (1:1000) (Sigma Aldrich, USA), or rabbit polyclonal antibodies to P19-TH (1:1000) (Thermo Fisher, USA), or to P40-TH (1:1000) (BioNovus, USA) in TBST with 1% BSA, or to P31-TH (1:1000) (Cell Signaling, USA) in TBST with 5% BSA, overnight at 4 °C. Then, the blots were washed in TBST, incubated with secondary horseradish peroxidase-conjugated anti-mouse or anti-rabbit IgG antibodies (Jackson ImmunoResearch Laboratories, USA) in TBST for 2 h and washed in TBST. The conjugated antibodies were visualized using enhanced chemiluminescence in 0.1 M Tris-HCl with 12.5 mM luminol, 2 mM coumaric acid, and 0.03% H_2_O_2_ (pH 8.5). The intensity of TH and P19-, P31-, and P40-TH protein bands was measured by densitometry using ImageLab software (Biorad, USA) and then were normalized to the Ponceau signal which is more stable than β-actin in the case of the neurodegeneration [34,35,36,37]. The results were expressed as relative optical density in the experimental groups compared with those in the saline injected group taken as 100%. Western blot of TH, P31-TH, P40-TH and P19-TH immunoreactivity and Ponceau staining in the striatum and in the SN are represented in the Figures S1 and S2.

### 2.5. Statistical Analysis

Data are presented as the group mean ± standard error of mean. The correspondence of the data to the normal distribution was checked using the Shapiro–Wilk test. The results were statistically processed with the GraphPad Prism 6.0 software package (GraphPad Software, USA) using one-way ANOVA and parametric Student’s *t*-test. *p* ≤ 0.05 was used everywhere as the significance criterion.

## 3. Results

### 3.1. The Concentration of Dopamine and Its Metabolites in the Striatum

**Figure 2:**
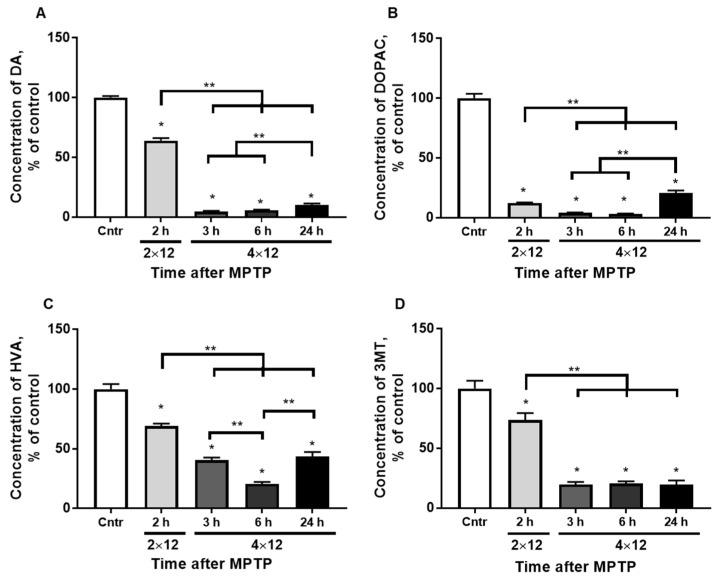
The concentration of DA (**A**), DOPAC (**B**), HVA (**C**), and 3MT (**D**) in the striatum of the control and 2 h after 2 × 12 mg/kg of MPTP and 3, 6, and 24 h after 4 × 12 mg/kg of MPTP. The results are presented as percentages of those of the control (100%). * *p* < 0.05 compared with the control (saline). ** *p* < 0.05 compared with selected MPTP groups.

The average concentration of DA in the striatum in control animals was 102.6 ± 0.8 pmol/mg (Figure 2A). The DA level decreased to 65.0 ± 2.1 pmol/mg 2 h after two MPTP injections. It was 4.8 ± 0.6 and 6.0 ± 0.4 pmol/mg 3 and 6 h after four MPTP injections, respectively, and after 24 h it was 10.5 ± 0.5 pmol/mg. 

A significant difference in the concentration of DA was observed between the experimental group with 2 h after the two MPTP injections and the groups with 3 and 6 h after the four MPTP injections.

The average concentration of DOPAC in the striatum in the control groups was 6.5 ± 0.2 pmol/mg (Figure 2B). The DOPAC concentration decreased to 0.7 ± 0.03 pmol/mg 2 h after two injections of MPTP. It was 0.3 ± 0.02 and 0.2 ± 0.01 pmol/mg 3 and 6 h after four MPTP injections, respectively, and after 24 h it was 1.3 ± 0.04 pmol/mg. A significant difference was observed between the experimental group with 2 h after two MPTP injections, and the groups with 3, 6, and 24 h after four MPTP injections as well as between the groups with 3, 6, and 24 h after the injections.

The average concentration of HVA in the striatum in the control groups was 7.7 ± 0.3 pmol/mg (Figure 2C). The concentration of HVA decreased to 4.0 ± 0.2 pmol/mg 2 h after two MPTP injections. It was 4.1 ± 0.6 pmol/mg 3 h after four MPTP injections, 1.8 ± 0.2 pmol/mg after 6 h, and 3.7 ± 0.1 pmol/ mg after 24 h. Significant differences were observed between the experimental group with 2 h after the two MPTP injections and the groups with 3, 6, and 24 h after four MPTP injections as well as between the groups with 3 and 6 h and with 6 and 24 h after injections.

The average concentration of 3MT in the striatum in the control groups was 2.1 ± 0.1 pmol/mg (Figure 2D). The 3MT level decreased to 1.4 ± 0.1 pmol/mg 2 h after two MPTP injections. The concentration of 3MT was 1.1 ± 0.6 and 0.5 ± 0.05 pmol/mg 3 and 6 h after four MPTP injections, respectively, and after 24 h it was 0.4 ± 0.02 pmol/mg. Significant differences were observed between the experimental group with 2 h after the two MPTP injections, and the groups with 3, 6, and 24 h after four MPTP injections.

### 3.2. Dopamine Turnover in the Striatum

**Figure 3:**
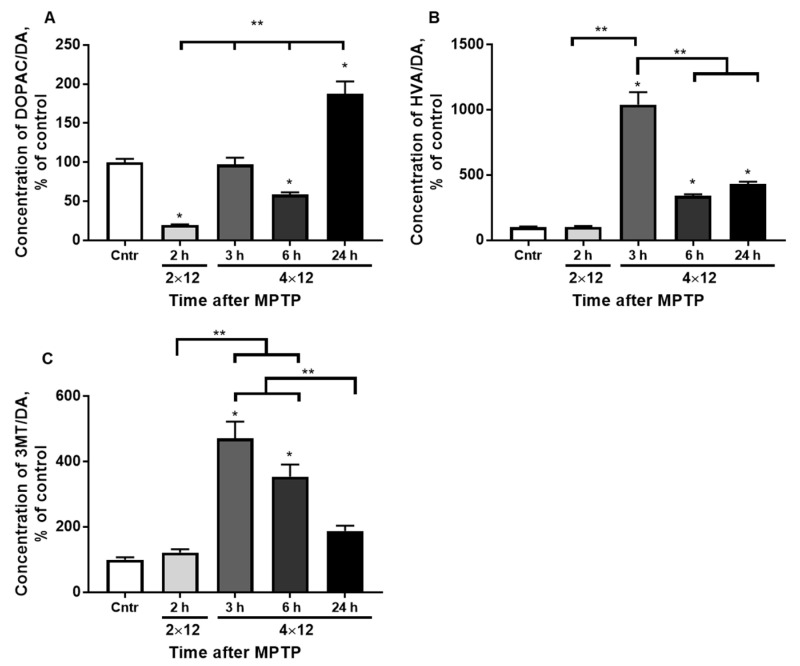
The DA turnover calculated by DOPAC/DA (**A**), HVA/DA (**B**), and 3MT/DA (**C**) in the striatum of the control and 2 h after 2 × 12 mg/kg of MPTP and 3, 6, and 24 h after 4 × 12 mg/kg of MPTP. The results are presented as percentages of those in the control (100%). * *p* < 0.05 compared with the control (saline). ** *p* < 0.05 compared with selected MPTP groups.

The DOPAC/DA ratio decreased to 19% and 51% 2 h after two MPTP injections and 6 h after four MPTP injections, respectively. The DOPAC/DA ratio was at the control level after 3 h, and after 24 h it increased to 184% (Figure 3A). Significant differences between all experimental groups were observed.

The HVA/DA ratio 2 h after two MPTP injections remained at the control level. The HVA/DA ratio was 1010% 3 h after four injections of MPTP, and it was about 400% after 6 and 24 h (Figure 3B). Significant differences were found between the experimental group collected 3 h after four MPTP injections and all the other experimental groups.

The 3MT/DA ratio remained at the control level 2 h after two MPTP injections. The 3MT/DA ratio was about 400% on average 3 and 6 h after four MPTP injections, and 184% 24 h after MPTP (Figure 3C). Significant differences were found between the experimental groups with 3 and 6 h after four MPTP injections and the groups with 2 h after two MPTP injections and 24 h after four MPTP injections.

### 3.3. The Concentration of L-DOPA in the Striatum and SN after Inhibition of AADC with NSD-1015

**Figure 4:**
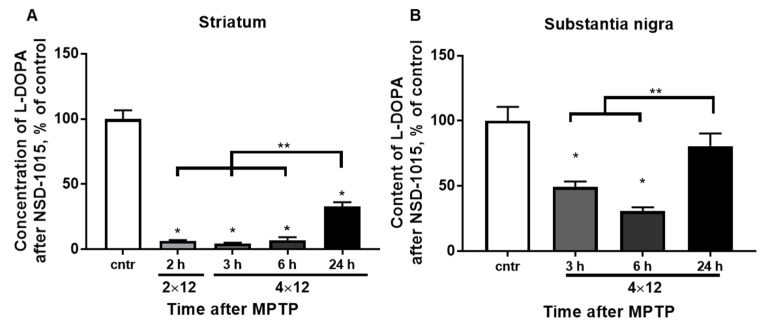
(**A**) The concentration of L-DOPA in the striatum upon inhibition of AADC by NSD-1015 (100 mg/kg 30 min before decapitation) of the control and 2 h after 2 × 12 mg/kg of MPTP and 3, 6, and 24 h after 4 × 12 mg/kg of MPTP. (**B**) The content of L-DOPA in the SN upon inhibition of AADC by NSD-1015 (100 mg/kg 30 min before decapitation) of the control and 3, 6, 24 h after 4 × 12 mg/kg of MPTP. The results are presented as percentages of those in the control (100%). * *p* < 0.05 compared with the control (saline). ** *p* < 0.05 compared with selected MPTP groups.

The average concentration of L-DOPA in the striatum in the control group 30 min after the administration of NSD-1015 at a dose of 100 mg/kg was 17.1 ± 1.4 pmol/mg (Figure 4A). The concentration of L-DOPA was 7.1 ± 0.3, 5.4 ± 0.8, and 7.2 ± 2.2% of that in the control 2 h after two MPTP injections, 3 and 6 h after four MPTP injections, respectively, and after 24 h it was 32.8 ± 3.4%. Significant differences were found between the experimental group with 24 h after the MPTP injections and all other experimental groups.

The average concentration of L-DOPA in the SN in the control group 30 min after the administration of NSD-1015 at a dose of 100 mg/kg was 11.5 ± 0.6 pmol (Figure 4B). The L-DOPA concentration was 49.3 ± 4.2 and 30.8 ± 2.8% of that in the control 3 and 6 h after four MPTP injections, respectively, and after 24 h, the L-DOPA concentration was at the control level. Significant differences were found between the experimental groups with 3 and 6 h and the group with 24 h after four MPTP injections.

### 3.4. The Content of TH and Its Phosphorylated Forms in the Striatum

**Figure 5:**
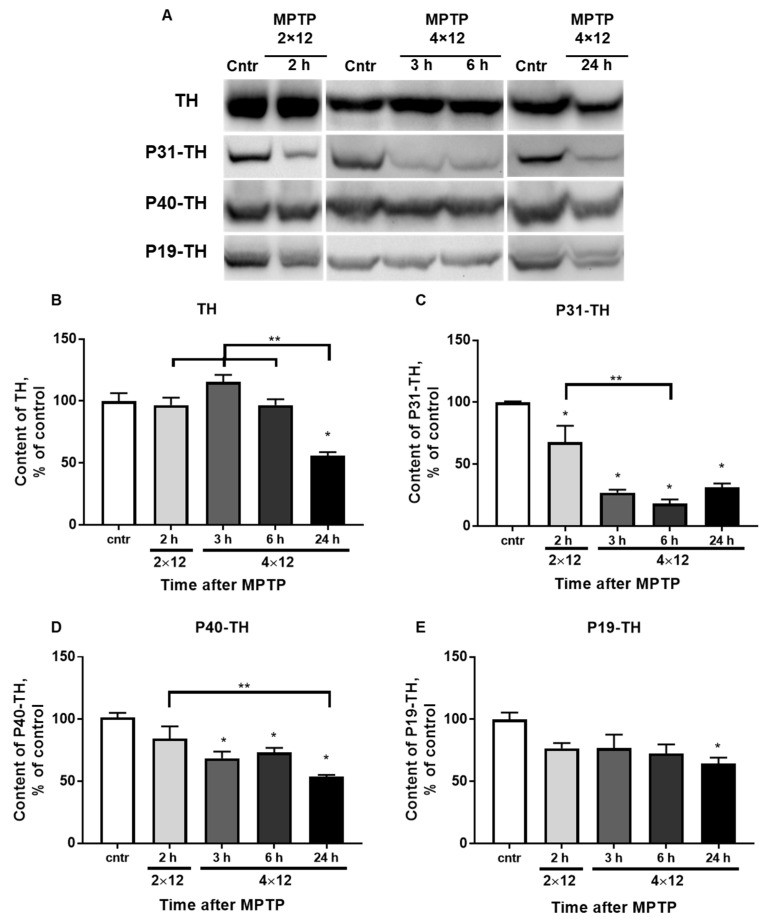
(**A**) Western blot representation of TH, P31-TH, P40-TH, and P19-TH immunoreactivity in the striatum of the control and 2 h after 2 × 12 mg/kg of MPTP and 3, 6, and 24 h after 4 × 12 mg/kg of MPTP. (**B**–**E**) Bar graph representation of TH (**B**), P31-TH (**C**), P40-TH (**D**), and P19-TH (**E**) in the striatum of the control (saline) and 2 h after 2 × 12 mg/kg of MPTP and 3, 6, and 24 h after 4 × 12 mg/kg of MPTP. The results are presented as percentages of those in the control (100%). * *p* < 0.05 compared with the control (saline). ** *p* < 0.05 compared with selected MPTP groups.

The total TH content in the striatum (Figure 5A,B) remained at the control level during the first 6 h after four MPTP injections and decreased by 43% after 24 h. Significant differences were found between the experimental group with 24 h after four MPTP injections and the groups with 2 h after two MPTP injections and with 3 and 6 h after four MPTP injections.

P31-TH content (Figure 5A,C) decreased by 50% compared with that of the control 2 h after two MPTP injections. The content of this phosphorylated form of TH was 25, 14 and 30% after 3, 6, and 24 h, respectively. Significant differences were found between the experimental groups collected 2 h after two MPTP injections and 6 h after four MPTP injections.

P40-TH content (Figure 5A,D) did not change compared with that of the control 2 h after two MPTP injections. It decreased by 30% 3 and 6 h after four MPTP injections, respectively, and by 45% after 24 h. Significant differences were found between the experimental group with 2 h after two MPTP injections and the group with 24 h after four MPTP injections.

P19-TH content (Figure 5A,E) did not change up to 6 h after four MPTP injections, and after 24 h it decreased to 65%.

### 3.5. The Content of TH and Its Phosphorylated Forms in the SN

**Figure 6:**
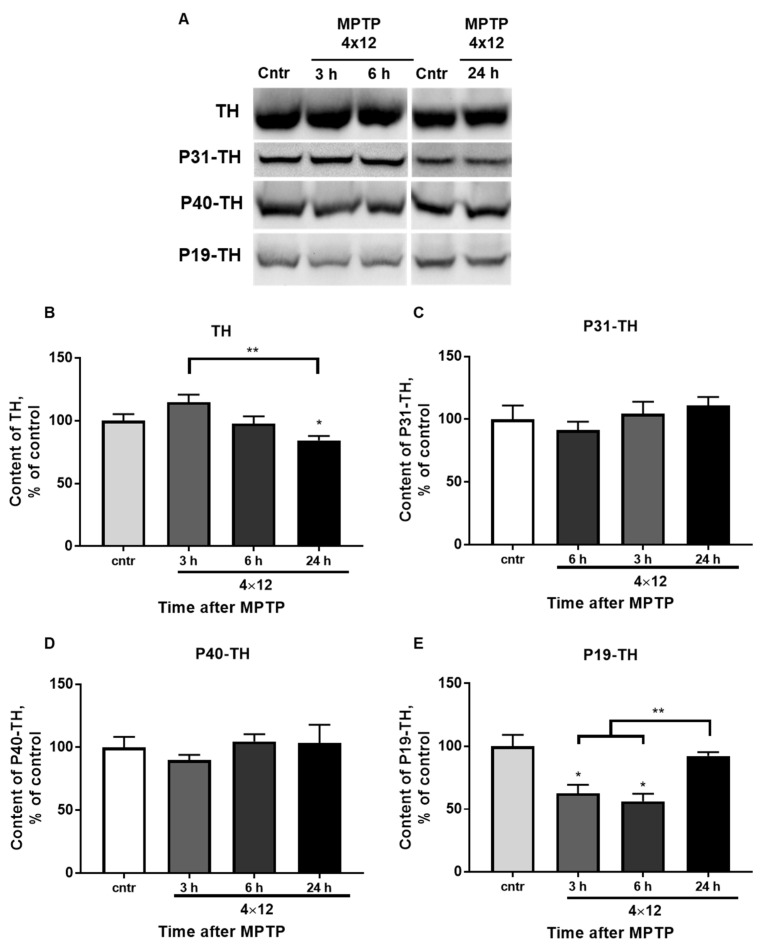
(**A**) Western blot representation of TH, P31-TH, P40-TH, and P19-TH immunoreactivity in the SN of the control and 3, 6, and 24 h after 4 × 12 mg/kg of MPTP. (**B**–**E**) Bar graph representation of TH (**B**), P31-TH (**C**), P40-TH (**D**), and P19-TH (**E**) in the SN of the control (saline) and 3, 6, and 24 h after 4 × 12 mg/kg of MPTP. The results are presented as percentages of those in the control (100%). * *p* < 0.05 compared with the control (saline). ** *p* < 0.05 compared with selected MPTP groups.

The content of total TH in the SN (Figure 6A,B) remained at the control level during the first 6 h after four MPTP injections. It decreased by 13% 24 h after four MPTP injections. Significant differences were found between the experimental groups with 3 and 24 h after four MPTP injections.

The content of P31-TH and P40-TH (Figure 6A,C,D) did not change in the studied intervals after MPTP administration.

The content of P19-TH (Figure 6E) decreased by 43 and 38% 3 and 6 h after four MPTP injections, respectively. The content of P19-TH was at the control level after 24 h. Significant differences were found between the experimental group with 24 h after four MPTP injections and the groups with 3 and 6 h after four MPTP injections.

## 4. Discussion

The prevailing number of studies use acute modeling of PD in mice, i.e., administration of MPTP within one day using one to four injections at doses ranging from 10 to 50 mg/kg [38]. Previously, an acute model of PD was developed in our laboratory with four-fold administration of 12 mg/kg of MPTP with a 2 h interval. For this model, threshold degradation of the nigrostriatal system with impaired motor behavior in mice was shown two weeks after neurotoxin administration [30]. This model, as well as similar ones, are commonly used to study neuroplasticity processes that develop in the presence of an emerging DA deficiency and loss of DA neurons [31,39,40].

**Figure 7:**
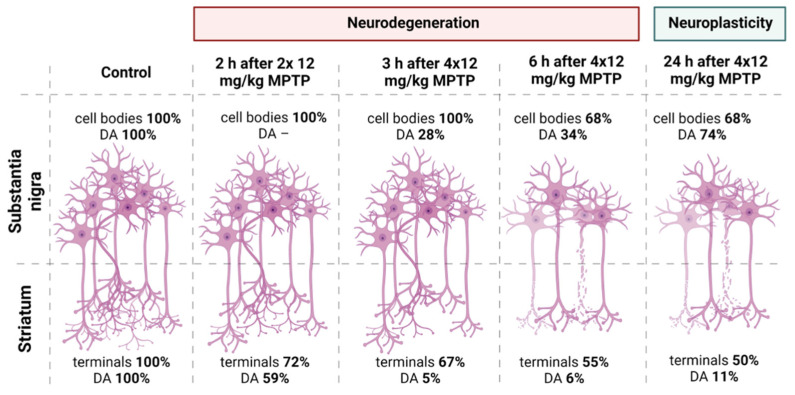
The quantity of DA neuron cell bodies and content of DA in the SN, the quantity of terminals of DA axons and DA concentration in the striatum in the control (saline) and during 24 h after 4 × 12 mg/kg of MPTP (the data adapted with permission from Ref. [29]). The figure was created using Biorender (www.biorender.com, the date of last access to the link is 14 June 2022).

We evaluated the period of degradation of the nigrostriatal system using the developed model [30] in order to study neurodegenerative processes. We demonstrated that the terminals of DA axons react earlier to the injection of a neurotoxin, and the period of their degradation lasts longer than the death of neuronal bodies in the SN. The degree of functional inhibition (decrease in DA concentration) in the striatum prevailed over the degree of functional inhibition in the SN. At the same time, in contrast to those in the striatum, surviving DA neurons in the SN demonstrated partial recovery of their functional state (Figure 7).

In this study, we evaluated DA synthesis during the period of neurodegeneration in the nigrostriatal system and during the first hours after its completion—the period of neuroplasticity. The key indicators used were the total content and activity of TH, as well as the content of its phosphorylated serine forms at positions 19, 31, and 40 (P19-, P31-, and P40-TH).

### 4.1. Dopamine Synthesis in the Substantia Nigra

The content of TH in a neuron body is the result of three processes—synthesis, degradation, and transportation along processes (axons, dendrites). According to the data obtained, the content of TH remained at the control level during 6 h after the last (fourth) injection of MPTP. At the same time, after 3 h, the number of neuron bodies did not change, and after 6 h, it decreased by 32%, i.e., in the surviving DA neurons, the content of TH increased compared to that in the control. The number of neuron bodies did not change, while the level of TH decreased by 13%, 24 h after the last injection. The discovered changes are not associated with a decrease in the content of TH in the neuron bodies (by IHC) [30]. This is probably partly due to the reduced activity of the ubiquitin-dependent system of protein proteolysis one day after a similar regimen of MPTP administration [41,42]. Probably, the observed decrease in the TH content reflects the restoration of anterograde protein transport along the fibers [43,44].

The TH activity decreased by 50–60% 3 and 6 h after the fourth injection of MPTP compared to the control level. The content of DA decreased by approximately the same amount [30]. This confirms a direct relationship between enzyme activity and neurotransmitter levels in SN. After MPP+ enters DA neurons via DAT, in addition to inhibition of the Mitochondrial Respiratory Complex I and initiation of oxidative stress, pumping of MPP+ (MPTP metabolite which is a toxin for DA neurons) into synaptic vesicles via VMAT2 begins where MPP+ competitively replaces DA [45,46,47,48,49,50]. Meanwhile, an increased level of cytosolic DA has not only a toxic effect on the DA neuron [50,51], but can also bind to the TH enzyme, inhibiting the latter [52,53].

Interestingly, in the same period (3 and 6 h after four injections of MPTP), a decrease in the content of TH phosphorylated by serine at position 19 (P19-TH) was shown. Dephosphorylation of P19-TH in the SN and the striatum is performed by protein phosphatase 2A (PP2A) [54]. An increase in the expression of α-synuclein, mitochondrial dysfunction, induction of oxidative stress, and apoptosis leads to an increase in the activity and/or content of PP2A [54,55]. In chronic modeling of PD by administration of MPTP, mice show an increase in the PP2A content in SN [56], and monkeys (Cynomolgus monkeys) demonstrate a decrease in PP2A activity [57]. However, in the above-mentioned studies, the content and activity of PP2A were evaluated after the completion of neurodegeneration and the onset of the development of neuroplasticity. We assume that under our experimental design, the decrease in P19-TH 3 and 6 h after four injections of MPTP was provoked by an increase in the activity and/or content of PP2A, which occurred because of mitochondrial dysfunction and induction of oxidative stress by MPP+.

During the first day after MPTP administration, correlations between TH activity and P19-TH content were observed. Both criteria were lowered 3 and 6 h after four injections of MPTP, and by 24 h, they were restored to the control level. The relationship between the activity of TH and P19-TH was not obvious. Moreover, according to the available data, the phosphorylated form does not directly affect the activity of TH *in vitro* and *in vivo* [22,27]. However, on the MN9D line (hybrid neuroblastoma-immortalized DA mesencephalic neurons from C57Bl/6 mice embryos), it was shown that P19-TH binds to chaperone proteins of the 14-3-3 family, which can lead to an increase in TH activity [58,59,60]. In our case, the dissociation of P19-TH and proteins 14-3-3 may occur, or this complex may bind to α-synuclein, which leads to a decrease in TH activity [61]. Unfortunately, no studies have been found evaluating the level of 14-3-3 proteins during degeneration in acute PD modeling in mice. At the same time, data on the absence of changes or even a decrease in the expression of the gene and protein of α-synuclein in SN in the first hours and days after a similar administration of MPTP [41,62,63,64] do not support the above-mentioned hypothesis.

According to the data on the stoichiometry of phosphorylated TH forms in rats, the content of P40-TH in SN is the lowest compared to other forms [65,66], and the absence of changes in its content during the first day after MPTP administration indicates its secondary role in determination of TH activity during the period of neurodegeneration and neuroplasticity [25,27]. We also found no changes in the P31-TH level in the SN on the first day after MPTP administration. However, during the period of neuroplasticity (seven days and later after MPTP or 6-OHDA administration), an increase in the content of this form of TH was shown [25,27].

Thus, it can be concluded that during the degradation of the nigrostriatal system and the period of neuroplasticity, the level of DA in the SN is determined by the activity of the TH enzyme, but not by its content. P19-TH plays a role in the establishment of enzyme activity; however, further studies are required to establish the mechanism of this regulation.

### 4.2. Dopamine Synthesis in the Striatum

In this study, the concentration of DA was re-determined in the striatum. The change in DA concentration was the same as in previous studies with a similar scheme of MPTP administration [29,30]. However, the use of Shimadzu equipment with high resolution (Shimadzu Corporation, Japan) made it possible to identify differences not only between the DA level 2 h after two MPTP injections and all other conditions, but also between 3, 6, and 24 h after four MPTP injections (Figure 2A).

The concentration of DA in the striatum is the result of three coupled processes: synthesis, degradation, and reuptake from the synaptic cleft into vesicles involving DAT and VMAT2 to reuse DA. Notably, in the normal striatum, DA reuptake prevails over its degradation [7]. An indicator of DA synthesis is the content and activity of TH, which together make it possible to calculate the specific activity of the enzyme as the ratio of TH activity to its content [14,15].

According to the data, the total content of TH in the striatum remained at the control level during the period of degradation of the DA axon terminals (up to 6 h after four MPTP injections), and after 24 h it decreased by 40%. It is likely that the observed decrease in the TH level was associated with the retrograde spread of the neurodegenerative process from the axon terminals further along the axons towards the neuron bodies. Changes in the content of total TH also correlated with the level of P19-TH; however, the presence of a causal relationship between these indicators was not obvious.

Changes in TH activity in the striatum, as well as in the SN, did not correlate with the total TH content. At the same time, TH activity decreased by 10 times 2 h after two injections of MPTP and remained at this level up to 6 h after four injections. After 24 h, the TH content and its homospecific activity partially restored (up to 30% and up to 50%, respectively) [14]. Such a significant decrease in enzyme activity during the first 6 h probably occurs by the same mechanism as in the SN. This refers to the inhibition of TH by DA, the level of which increases in the cytosol after the entry of MPP+ into the synaptic terminals of DA axons. This is confirmed by a gradual decrease in the concentration of DA in the striatum, which “lags behind” the decrease in TH activity, as well as an increase in DA turnover after 3 h in such criteria as 3MT/DA and HVA/DA.

A more significant decrease in TH activity in the striatum than in the SN is associated with a difference in the level of DA in these two structures. The content of DA in the striatum is 650 times higher than in the SN (unpublished data), while the difference in TH content is not so high (three-to-five times higher in the striatum) [65,66].

A partial recovery of DA concentration and TH activity 24 h after four injections of MPTP is associated with the onset of the development of reparative processes, primarily the uptake of the neurotransmitter into synaptic vesicles that have already been released from MPP+. According to Fornai, 12 h after MPTP administration (4 × 20 mg/kg), MPP+ content in the striatum is 10% of the peak value, which occurs 1–2 h after MPTP administration [67]. This hypothesis is also confirmed by the gradual return of DA turnover in the striatum to the control value (HVA/DA and 3MT/DA).

Another mechanism that negatively affects TH activity is dephosphorylation. According to our data, 2 h after two MPTP injections, the content of P31-TH in the striatum decreased significantly. There is a subsequent decrease in contents of both this phosphorylated form of TH and P40-TH 3, 6, and 24 h after four MPTP injections. The results obtained are also consistent with data obtained 2 h after 15 mg/kg of MPTP and 9–16 days after 6-OHDA administration [26,27]. Considering that the amount of P31-TH is normally higher than that of P40-TH, and that the content of P31-TH after the induction of neurodegeneration decreases to a greater extent than that of P40-TH does, it can be concluded that the content of P31-TH to a greater extent determines the activity of TH in the striatum during the degradation of the nigrostriatal system. In addition to TH dephosphorylation during this critical period, an increase in enzyme nitration was shown with a similar scheme of MPTP administration (4 × 20 mg/kg), which also has an inhibitory effect on its activity [68].

Thus, a decrease in TH activity during the first 6 h after the last injection of MPTP is associated with its dephosphorylation at serine at position 31 (P31-TH) and partially at position 40 (P40-TH), as well as with cytosolic DA inhibition.

A partial recovery of TH activity and DA concentration was shown 24 h after four MPTP injections. Even though there were no significant differences between the content of P31-TH 6 and 24 h after four MPTP injections, the average values differed by a factor of two. Perhaps, if we observed a longer period after the administration of MPTP, when neuroplasticity continued to develop, significant differences would be shown. This would confirm the importance of the role of P31-TH for the activity of this enzyme not only during the period of degradation of the nigrostriatal system but also during neuroplasticity.

Thus, it was shown that TH activity in the striatum during the period of degradation of the nigrostriatal system and neuroplasticity was not determined by the total content of the enzyme, but largely depended on the level of P31-TH and, to a lesser extent, on P40-TH, as well as on the level of cytosolic DA. The role of P19-TH in determination of the total TH level in the retrograde degradation of DA fibers should be defined in future studies.

## 5. Conclusions

An analysis of DA synthesis in the nigrostriatal system during its degradation and in the first hours after its completion, i.e., at the beginning of neuroplasticity, in a model of the early clinical stage of PD, showed that:

1. DA content in the SN and the striatum did not depend on TH content but correlated with enzyme activity.
2. TH activity did not depend on the total protein content either in the SN or in the striatum.
3. TH activity in the SN was determined by the content of P19-TH; TH activity in the striatum was determined by P31-TH and P40-TH (to a lesser extent).

The data obtained indicated different regulation of DA synthesis in DA neuron bodies and their axon terminals. These data should be taken into account for the further development of symptomatic pharmacotherapy aimed at increasing TH activity.

## Data Availability

The data presented in this study are available on request from the corresponding author. The data are not publicly available due to legal issues.

## Supplementary Materials

The following supporting information can be downloaded at: https://www.mdpi.com/article/10.3390/brainsci12060779/s1, Figure S1: Western blot representation of TH, P31-TH, P40-TH and P19-TH immunoreactivity and Ponceau staining in the striatum of the control, 2 h after 2 × 12 mg/kg of MPTP and 3, 6, 24 h after 4 × 12 mg/kg of MPTP; Figure S2: Western blot representation of TH, P31-TH, P40-TH and P19-TH immunoreactivity and Ponceau staining in the SN of the control and 3, 6, 24 h after 4 × 12 mg/kg of MPTP.
