# Supplementary material for: Changes in Tyrosine Hydroxylase Activity and Dopamine Synthesis in the Nigrostriatal System of Mice in an Acute Model of Parkinson’s Disease as a Manifestation of Neurodegeneration and Neuroplasticity
Source: Brain Sci. 2022 Jun 14;12(6):779. doi: 10.3390/brainsci12060779 (PMC9221104; doi:10.3390/brainsci12060779)
Supplement: Supplementary file 1 [file brainsci-12-00779-s001.zip › brainsci-1748654-supplementary.pdf]

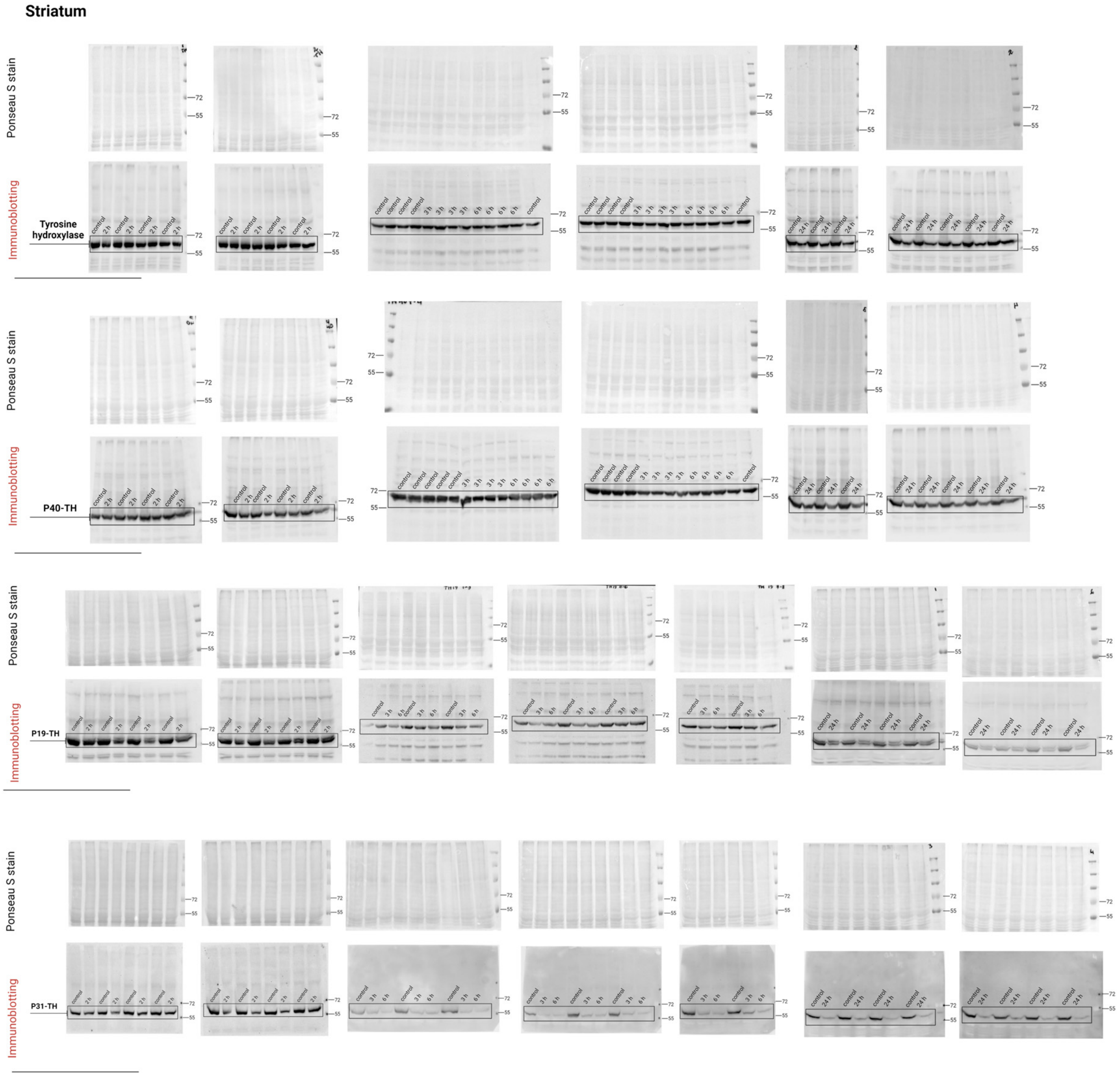

Figure S1. Western blot representation of TH, P31-TH, P40-TH and P19-TH immunoreactivity and Ponceau staining in the striatum of the control, 2 h after 2 × 12 mg/kg of MPTP and 3, 6, 24 h after 4 × 12 mg/kg of MPTP.

Substantia Nigra

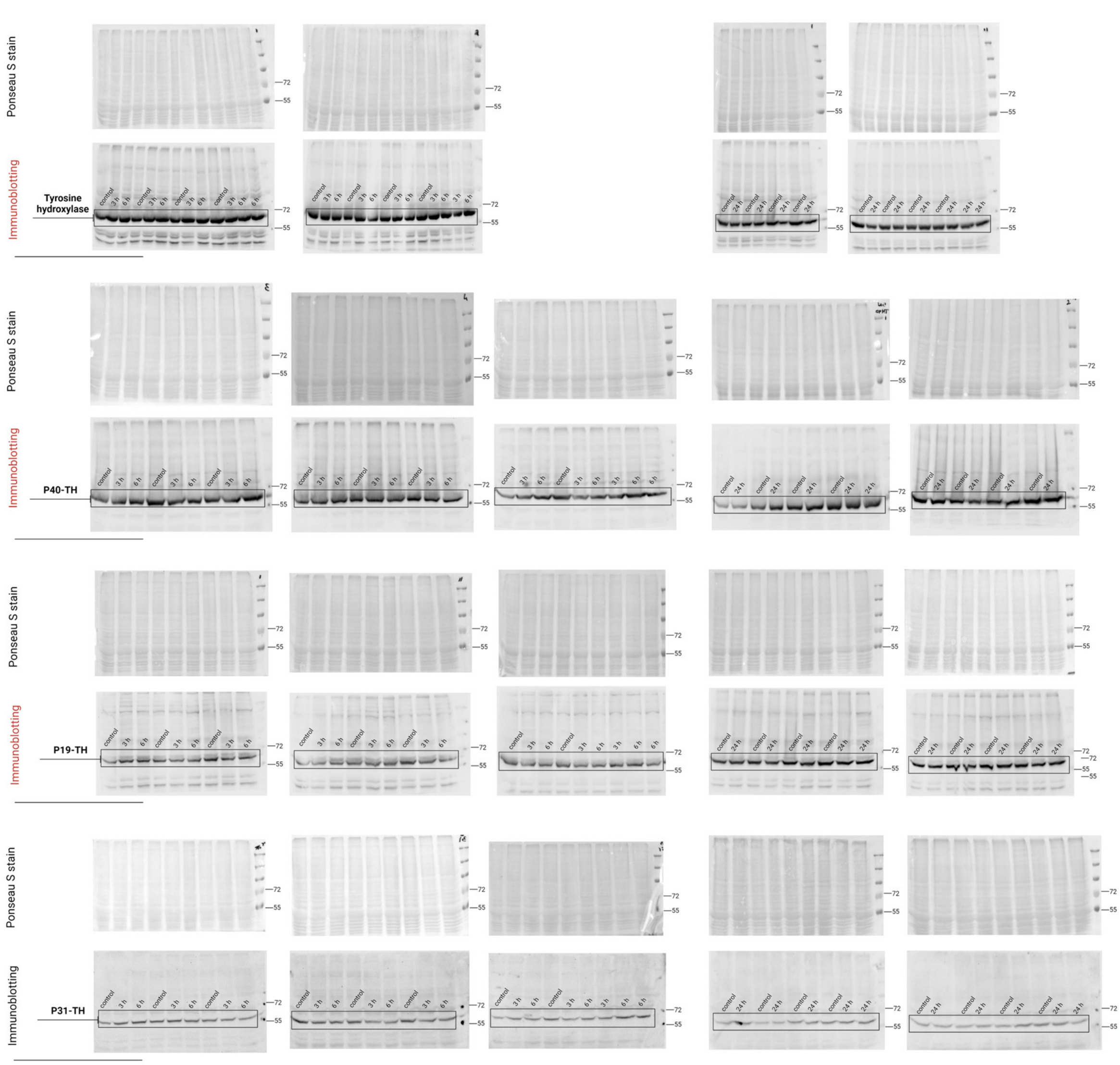

Figure S2. Western blot representation of TH, P31-TH, P40-TH and P19-TH immunoreactivity and Ponceau staining in the SN of the control and 3, 6, 24 h after 4 × 12 mg/kg of MPTP.
